# Enhancing dielectric permittivity for energy-storage devices through tricritical phenomenon

**DOI:** 10.1038/srep40916

**Published:** 2017-01-18

**Authors:** Jinghui Gao, Yan Wang, Yongbin Liu, Xinghao Hu, Xiaoqin Ke, Lisheng Zhong, Yuting He, Xiaobing Ren

**Affiliations:** 1 State Key Laboratory of Electrical Insulation and Power Equipment and Multi-disciplinary Materials Research Center, Frontier Institute of Science and Technology, Xi’an Jiaotong University, Xi’an, 710049, China; 2 Center of Microstructure Science, Frontier Institute of Science and Technology, Xi’an Jiaotong University, Xi’an, 710049, China; 3 Ferroic Physics Group, National Institute for Materials Science, Tsukuba, 305-0047, Ibaraki, Japan

## Abstract

Although dielectric energy-storing devices are frequently used in high voltage level, the fast growing on the portable and wearable electronics have been increasing the demand on the energy-storing devices at finite electric field strength. This paper proposes an approach on enhancing energy density under low electric field through compositionally inducing tricriticality in Ba(Ti,Sn)O_3_ ferroelectric material system with enlarged dielectric response. The optimal dielectric permittivity at tricritical point can reach to *ε*_r_ = 5.4 × 10^4^, and the associated energy density goes to around 30 mJ/cm^3^ at the electric field of 10 kV/cm, which exceeds most of the selected ferroelectric materials at the same field strength. The microstructure nature for such a tricritical behavior shows polarization inhomogeneity in nanometeric scale, which indicates a large polarizability under external electric field. Further phenomenological Landau modeling suggests that large dielectric permittivity and energy density can be ascribed to the vanishing of energy barrier for polarization altering caused by tricriticality. Our results may shed light on developing energy-storing dielectrics with large permittivity and energy density at low electric field.

Energy storage, referring to the capture of energy generated at one time and consumed at a later time, is one of vital technologies for the rational utilization of energy, aiming to meet the challenge of depletion of fossil fuels and global warming. At present, electrical power is considered as the backbone of modern industry and the society, and the storage of electric energy provides a direct approach to manipulate and efficiently utilize the power source. The dielectric material is capable of storing the electric energy due to its polarization in the presence of external electric field, causing positive charge to store on one electrode and negative charge on the other^1^. Admittedly, the electrochemical devices (e.g. rechargeable batteries, supercapacitors, fuel cells etc.) exhibit larger energy density compared with dielectrics^2^. However, the dielectric energy-storing devices enable faster delivery of energy (i.e. shorter charge or discharge time), and thus can be found promising applications on hybrid electric vehicles, portable electronic devices as well as power pulse devices^3^.

The electrostatic energy density that stored in dielectrics can be calculated by the equation 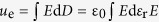, where *E* is the static electric field strength and *ε*_r_ is the relative dielectric permittivity. These two crucial parameters *E* and *ε*_r_ are decisive for the level of energy density for dielectric devices. Intensive investigations have been performed on the application of energy storage devices at high electric field^3^,^4^, which requires high breakdown strength for dielectrics. For example, owing to the high level of breakdown strength (*E*_b_), the polymer solid insulation dielectrics^5^,^6^ (e.g. PVDF copolymers^3^,^7^,^8^ and associated composite materials^9^,^10^,^11^,^12^) have been triggering tremendous research interests on achieving large energy density in the high electric field region (*E* ≈ 6000 kV/cm)^3^. However, the raising up of electric field strength is challenging the supporting insulation system, which may limit its applications on miniaturized equipments and portable or wearable electronic devices with high level of integration. Hence, it is desirable to develop energy-storing devices at finite electric field strength with comparable larger energy density. It should be noticed that as the other crucial factor for energy density, the dielectric permittivity is also vital on the performance of energy-storing devices in particular at finite field strength. Therefore, it is crucial to enhance the permittivity of dielectric materials for energy storage applications which are utilized in the low field strength region. We also notice that not all the high-permittivity materials (e.g. CaCu_3_Ti_4_O_12_ system with *ε*_r_ > 50000^13^,^14^,^15^,^16^) are suitable for energy storage application, because they are required to withstand considerable voltage and exhibit low dielectric loss. Therefore, the scope of this paper will focus on the high-permittivity ferroelectric material with relatively low conductivity.

## Results and Discussion

**Figure 1:**
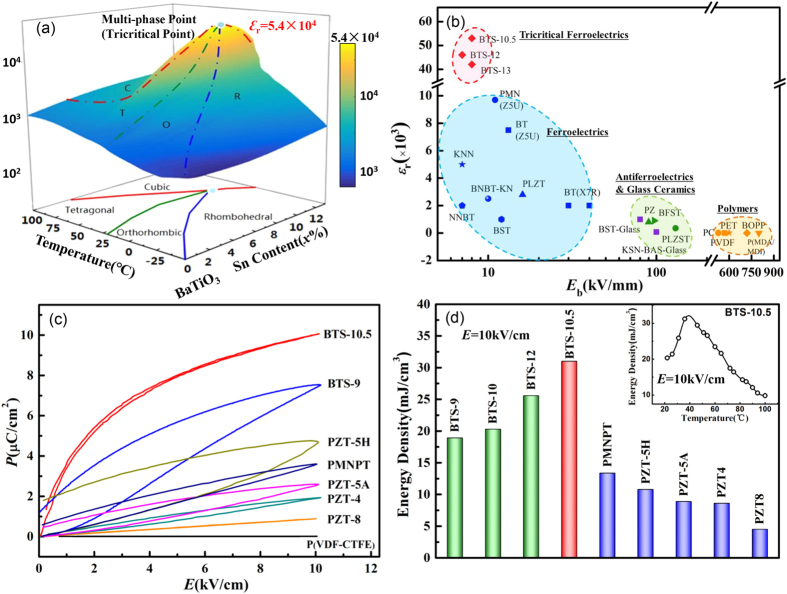
(**a**) The dielectric permittivity (*ε*_r_) distribution on the phase diagram of Ba(Ti_1-*x*%_Sn_*x*%_)O_3_ (BTS), and the maximum value can reach to 5.4 × 10^4^ at the multi-phase point which is also a tricritical point. (**b**) The locations for a series of materials categories on the dielectric permittivity(*ε*_r_)-breakdown strength (*E*_b_) plot (reproduced from refs 13, 14, 15, 16, 17, 18, 19, 20, 21, 22, 23), and the tricritical BTS materials at high permittivity region may result in a large energy storage properties at low electric field. (**c**) The polarization(P)-electric field (E) hysteresis loops for tricritical-point-nearby BTS-*x* and the other ferroelectric materials at low electric field region with *E* < 10 kV/cm. (**d**) The energy densities at 10 kV/cm for different material systems calculated from P-E loops. And the tricriticial ferroelectric BTS-10.5 exhibits the largest energy density of 31 mJ/cm^3^ at 10 kV/cm. The temperature variation of energy density for BTS-10.5 has been shown in the inset.

Here, by examining the dielectric permittivity distribution on the phase diagram of Sn doped barium titanate Ba(Ti_1-x%_Sn_x%_)O_3_ (abbreviated as BTS-*x*) ferroelectric system, we propose a novel approach to enlarge the dielectric permittivity as well as energy density through finding the composition-induced tricritical point. Figure 1(a) shows the temperature-composition phase diagram for Ba(Ti_1-x%_Sn_x%_)O_3_ (BTS-*x*) material system, which has been obtained by monitoring the dielectric anomalies at transition temperatures in combination with detecting crystal structures using X-ray diffraction method. It can be seen that as the increase of Sn doping concentration, one paraelectric phase (cubic) and three ferroelectric phases (of tetragonal, orthorhombic, rhombohedral symmetry) converge into a multi-phase point (with BTS-10.5 composition at 37 °C). We then investigated the dielectric permittivity variation with temperature and composition and thus depicted the distribution of *ε*_r_ on such a phase diagram as shown in the three-dimensional surface graph of Fig. 1(a). It is found that strong dielectric response with *ε*_r_ = 5.4 × 10^4^ appears in BTS-*x* material system, which is 5 times as high as that of pure BaTiO_3_. Other parameters such as loss tangent are also shown in Table 1. The optimal permittivity has been found on the multi-phase point, which will be verified as a tricritical point in the later part of this paper. We then depicted the BTS-*x* (x = 10.5, 12, 13) close to tricritical point (nominated as tricritical ferroelectrics) on the *E*_b_-*ε*_r_ plot and compared it with a series of other energy storage material systems^17^,^18^,^19^,^20^,^21^,^22^,^23^,^24^,^25^,^26^,^27^ in Fig. 1(b). It can be seen that tricritical ferroelectrics occupy high permittivity region with *E*_b_ < 10 kV/mm, and can be expected to have good energy-storing performance at low electric field strength. We then explore the energy storage properties for BTS-*x* and other material systems through measuring the polarization (*P*)-electric field (*E*) hysteresis loop. Although a figure of merit 1/2*ε*_0_*ε*_r_*E*_b_^2^ is always used to qualify the energy density, it is only applicable to the linear capacitance with the invariant *ε*_r_. We notice that the dielectric permittivity for ferroelectric materials decrease with the increase of external electric field, thereby we used P-E loop measurement to evaluate the energy density that stored in our materials. As shown in Fig. 1(c), BTS-10.5 at its tricritical point (37 °C) exhibits a larger polarization (*P* > 10 μC/cm^2^) even at low electric field (*E* = 10 kV/cm), which exceeds other selected ferroelectric systems. Moreover, the energy densities for different materials have been calculated from the integral of *P-E* curves, and the tricritical ferroelectrics show comparable higher energy density (*u*_e_ ≈ 30 mJ/cm^3^) at low field of *E* = 10 kV/cm as shown in Fig. 1(d). Although the energy density varies with temperature (Δ*u*_e_ = 10 mJ/cm^3^ from 20 °C to 70 °C) and its stability needs to be further improved, our investigation still provides an effective approach on achieving higher energy density at low electric field through compositional inducing tricritical phenomenon in ferroelectric material.

The large dielectric response in the multiphase coexisting point can be understood by considering the contributions of dielectric activities using Rayleigh analysis^28^,^29^,^30^,^31^,^32^,^33^,^34^,^35^,^36^,^37^. It is well-known that the dielectric response consists of two dielectric activities, i.e. intrinsic and extrinsic contributions. The intrinsic contribution refers to the dielectric activity produced by lattice deformation under electric field. In contrast, the extrinsic contribution reflects the dielectric response induced by the movement of interfaces in the material, e.g. phase boundaries or domain walls. These dielectric contributions can be analyzed by detecting the polarization-electrical field (*P-E*) hysteresis curves under subswitching conditions in low field regime without the changes on domain structure or domain wall density. In general, the corresponding *P-E* hysteresis loop can be described by Rayleigh relationship as follows:


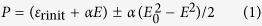



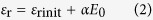


**Figure 2:**
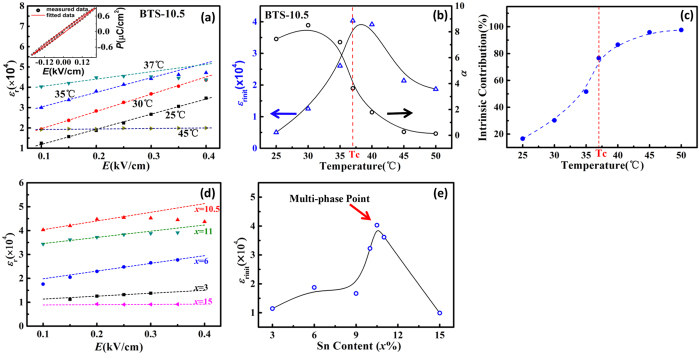
Rayleigh analysis for BTS-*x* material system. (**a**) Through low-field *P-E* hysteresis loop measurement (an example in the inset), the changes of dielectric permittivity as a function of electric field have been depicted for a series of temperatures for BTS-10.5, from which the intrinsic and extrinsic contribution can be evaluated. (**b**) Temperature-dependence of ε_init_ (intrinsic) and α (extrinsic) for BTS-10.5. (**c**) The percentage of intrinsic contribution for BTS-10.5 as a function of temperature, showing that the intrinsic contribution is the dominant dielectric activity at the multi-phase point. (**d**) Changes of dielectric permittivity as a function of electric field for different compositions at their individual Curie temperatures. (**d**) The composition-dependence of intrinsic coefficient ε_init_, indicating that the large dielectric response at multi-phase point can be ascribed to the maxima of intrinsic dielectric activity due to phase transition.

Here *E* is strength of the applied electric field with maximum value of *E*_0_. *P* refers to the polarization which changes with external electric field. The coefficient *ε*_rinit_ describes the intrinsic dielectric activity caused by lattice deformation which always shows non-hysteretic reversible dielectric response. On the other hand, the coefficient *αE*_0_, which reflects the hysteretic part of dielectric response, describes the extrinsic effect induced by the domain wall motion or phase boundary motion. Hence, we can evaluate the level of intrinsic and extrinsic dielectric contribution through the Rayleigh analysis. The inset of Fig. 2(a) shows the P-E hysteresis loop for optimal composition Ba(Ti_1-*x*%_Sn_*x*%_)O_3_ (*x* = 10.5) at the field amplitudes of 0.2 kV/cm at 37 °C. The measured curve can be well fitted by Rayleigh relation suggesting that the field strengths are within the Rayleigh region with subswitching condition for domain structure. We then measured the *P-E* loop for a series of electric field strength amplitudes. And Fig. 2(a) shows the electric field dependence of permittivity *ε*_r_ calculated by dividing the electric field amplitude into the maximum polarization value. It can be seen that the *ε*_r_ shows the linear relationship with *E*, which agrees with Rayleigh relationship in Eq. (2), and hence the intrinsic and extrinsic coefficients *ε*_rinit_ and *α* can be obtained through fitting. We then measured *P-E* hysteresis loop and calculated *ε*_rinit_ and *α* coefficients of BTS-10.5 for different temperatures across *T*_C_. And the associated temperature-dependent of ε_rinit_ and α have been shown in Fig. 2(b). It can be seen that with the increase of temperature the intrinsic coefficient *ε*_rinit_ for BTS-10.5 first increases, and then decreases on further heating, producing a peak value at *T*_C_. On the other hand, the extrinsic coefficient *α* decreases when temperature increases up to *T*_C_. Figure 2(c) shows the percentage for intrinsic contribution of BTS-10.5 calculated by *ε*_rinit_/(*ε*_rinit_+*αE*_0_), It can be seen that the level for intrinsic contribution has been largely enhanced in the vicinity of *T*_C_, which occupies more than 80% of dielectric contribution. It can thus be concluded that intrinsic dielectric response is the major contribution for strong dielectric response close to *T*_C_. We further compare the level of intrinsic dielectric contribution for BTS system with different compositions at their individual T_C_. The corresponding *P-E* hysteresis loops for a series of compositions have been measured, from which the *ε*_r_–*E* curves of different composition have been depicted (Fig. 2(d)). Fitted by Rayleigh relation, the intrinsic coefficient *ε*_rinit_ has been shown in Fig. 2(e) as a function of composition. It can be seen that the maximum value for *ε*_rinit_ appears at the *T*_C_ of BTS-10.5, which is the multi-phase point for BTS material system. Hence, it indicates that the reason for strong dielectric response at multi-phase point is mainly caused by intrinsic dielectric response which may be enhanced by phase transition.

**Figure 3:**
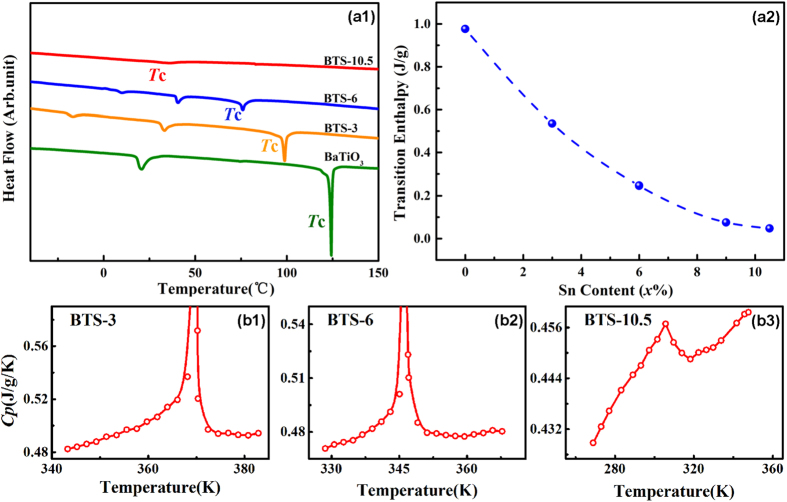
Thermal analysis of BTS-*x* material system. (**a1**) DSC heat flow curves for a series of compositions for BTS-*x* material system. (**a2**) The composition-dependence of transition enthalpy calculated from (a1) shows that transition enthalpy gradually reduce to a low level approaching zero, indicating a tricritical behavior for the multi-phase point for x = 10.5. (**b1**–**b3**) The specific heat-temperature (*C*_p_-*T*) curves for BTS-*x* system. The specimens of *x* = 3 and 6 exhibit divergent peak reflecting the first-order transition behavior. For the composition *x* = 10.5, however, it shows a λ-shaped specific peak with discontinuity, indicating that the first-order transition changes into the second-order at this point, i.e. tricritical point.

In order to further detect the phase transition behavior for such a multi-phase point, we perform thermal analysis for BTS-*x* ceramics with a series of compositions (*x* = 0~10.5), including the latent heat (transition enthalpy) and specific heat measurement. As it is known, the sudden change of the spontaneous polarization at a first-order transition gives rise to latent heat as well as divergent specific heat at its Curie temperature. On the other hand, tricritical behavior refers to a special thermodynamic condition where first-order transition changes into second-order^38^,^39^,^40^,^41^,^42^,^43^, and it thus involves discontinuous change of specific heat owing to the discontinuity in polarization^44^. Figure 3(a1) displays the heat flow curves for BTS-*x* specimens around their individual Curie temperatures measured by a differential scanning calorimeter (DSC). The temperature-dependence of heat flow curves for different-composition specimens are shown simultaneously. It can be seen that undoped BaTiO_3_ exhibits apparent first order transition characteristic with a sharp heat flow peak at *T*_C_ = 121 °C. However, as the concentration of Sn dopant increases, the heat flow peaks on Curie temperatures are suppressed so that the peak height is lowered. And when the composition changes to the multi-phase point (*x* = 10.5), the heat flow peak becomes nearly invisible, which suggests that the phase transition changes into second-order. We then obtained the latent heat (transition enthalpy) from the integral of heat flow curves at the peak regions around their individual Curie temperatures. The results are shown in Fig. 3(a2) as a function of composition. It can be seen that with the increase of Sn concentration, the transition enthalpy at Curie temperature decreases gradually. And when the composition goes to the multi-phase point, the transition enthalpy approaches to zero indicating that first order transition changes to the near second order. Hence, such a multi-phase point exhibits nearly tricritical behavior.

Moreover, the temperature-dependence of specific heat for BTS-*x (x* = 3, 6, 10.5) have been measured across their own Curie temperatures by using a physical property measurement system (PPMS). The specific heat measurement results of BTS-*x (x* = 3, 6) have been displayed in Fig. 3(b1),(b2) respectively. It can be seen that they show similar feature that the specific heat-temperature curves are divergent at their own Curie temperatures, which suggests a first-order transition nature for ferro-para transition. In contrast, BTS-10.5 shows a quite different specific heat characteristic. As shown in Fig. 3(b3), the specific heat curve for *x* = 10.5 exhibits a unique λ-shape curve: the curves at ferroelectric temperature region and paraelectric temperature region change continuously with temperature, but there is a sudden drop around Curie temperature, which reflects a discontinuous change of specific heat indicating a second-order transition behavior. Therefore, the specific heat curve for BTS-10.5 provides an evidence that the first-order transition goes into second-order (or weak first-order), and the multi-phase point exhibits tricritical phenomenon, which may be responsible for strong dielectric response as well as large energy density at low electric field.

**Figure 4:**
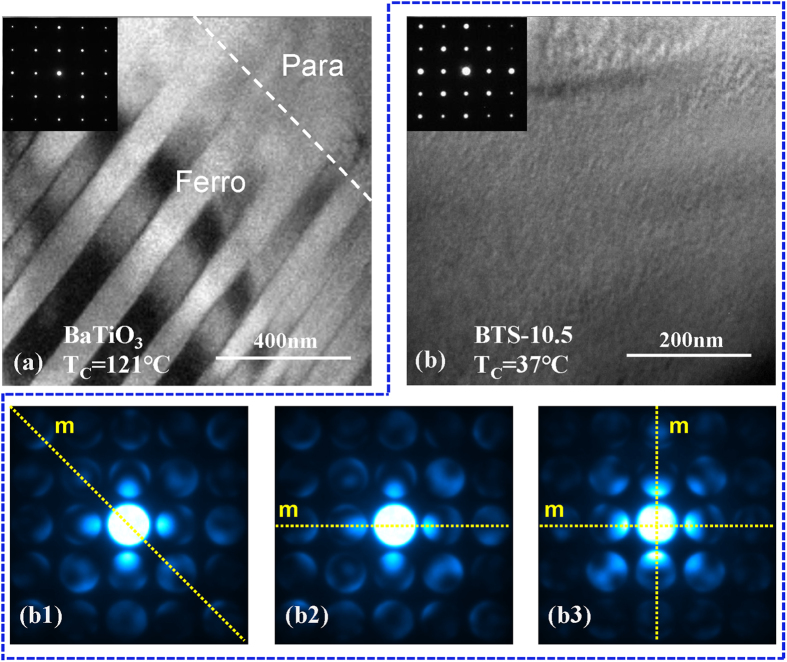
TEM bright field images for (**a**) undoped BaTiO_3_ at *T*_C_ showing clear para-ferro phase boundary and (**b**) BTS-10.5 at *T*_C_ (i.e. tricritical point) showing the mottled nanodomain pattern. (**b1–b3**) The CBED patterns of three neighboring locations for tricritical specimen, and the diverse patterns with different diffraction symmetries suggest the polarization inhomogeneity in nanometer scale.

What is the microstructure characteristic for multi-phase point showing tricriticality and how does it relate to large dielectric response and energy density? In order to answer the above-mentioned questions, we examined the domain structure of the tricritical point for BTS-*x* system via transmission electron microscope (TEM) observation. To make a comparison, we first performed the TEM observation for undoped BaTiO_3_ showing typical first order transition. Figure 4(a) shows the bright field TEM image for domain structure of BaTiO_3_ ceramic around Curie temperature with the beam incidence of [001]. It can be seen that the microstructure can be clearly separated into the ferroelectric portion with stripe domain pattern and paraelectric portion without domain. These two portions are bordered by a very apparent interphase boundary shown by the dashed line of Fig. 4(a). And such a microstructural feature reveals a phase coexisting of first order transition nature at the ferro-para transition temperature of BaTiO_3_, which is well-known in ferroelectrics. In contrast, the microstructure for BTS-10.5 ceramic at tricritical point exhibits distinguished feature. Unlike the undoped BaTiO_3_ with clear interphase boundary, the tricritical point specimen BST-10.5 exhibits a mottled microstructure shown in Fig. 4(b), which has numerous domains formed with the size of nanometer scale. Previous Brillouin scattering and relevant studies by S. Kojima *et al*.^45^ and T. H. Kim *et al*.^46^ suggest that the tricritical transition is accompanied by the formation of nano-regions in a parallel ferroelectric system Pb(Sc_1/2_Nb_1/2_)O_3_-PbTiO_3_ and PZT single crystal, which coincides with our TEM result for the tricritical point of BTS*-x* system. It should be noticed that such a nanodomain microstructure in BTS-*x* system is uniform in the grain, which differs from that of CaCu_3_Ti_4_O_12_ system exhibiting spatial defect inhomogeneity between grain and grain boundary and forming a internal barrier layer structure^14^. Therefore, such two material systems have different mechanisms for dielectric permittivity enhancement.

Moreover, in order to further detect the crystal structure for these nano-regions, we performed convergent beam electron diffraction (CBED) observation in TEM with a minuscule convergent electron-beam probe, and the CBED diffraction patterns with [001] beam incidence have been shown in the Fig. 4(b1)–(b3). According to the phase diagram in Fig. 1(a), four crystal symmetries, including three ferroelectric phases (T, O, R) and one paraelectric phase (C), are possible to appear depending on its temperature/composition conditions. Figure 4(b1)–(b3) show the diffraction patterns for the tricritical point of BTS-10.5 (37 °C), which has been taken from three neighboring sampling points within the same grain. It can be seen that the diffraction symmetries for these points differs with each other: (1) Fig. 4(b1) displays a CBED pattern with mirror plane along (110), which indicates rhombohedral or orthorhombic crystal symmetry. (2) Figure 4(b2) shows a CBED pattern with (010) mirror plane, and therefore suggests tetragonal crystal symmetry. (3) The pattern in Fig. 4(b3) exhibits 4 mm diffraction symmetry, and it is possible to be responsible for either cubic symmetry or tetragonal symmetry. Hence, the multi-phase point manifests itself as numerous nanoregions with polarization inhomogeneity in microstructure. Such a microstructure feature may be caused by the tricriticality of multi-phase point, which leads to low polarization anisotropy. And it may enable the adjacent nanoregions alter to each other very easily, and thus facilitate a large dielectric response in the presence of external electric field. Hence, large dielectric permittivity as well as energy density at low field can be expected from such a tricritical ferroelectric materials.

Our reported large dielectric permittivity can be explained by a Landau-type modeling regarding the thermodynamically special tricritical phenomenon. According to the classical theory of ferroelectrics, Gibbs free energy of the system can be expanded into a polynomial with respect to polarization *P* = (*P*_1_, *P*_2_, *P*_3_). Here we employ a sixth-order polynomial and omit the high-order terms, and the Gibbs free energy *G* can be written as follows^47^:


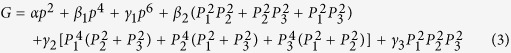


Here *α*_*i*_, *β*_*i*_, and *γ*_*i*_ are the expansion coefficients which also vary with the temperature *T* and composition *x*, and such changes on the coefficients *β*_2_, *γ*_2_ and *γ*_3_ determine the polarization anisotropy and the phase stability of different ferroelectric phases (tetragonal, orthorhombic and rhombohedral). The tricriticality of the multi-phase point, evidenced by our thermal analysis results, can guarantee a special thermodynamic condition with 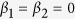 at the tricritical point. And by further tuning the temperature and composition dependency of the relevant coefficients, a phase diagram can be produced which coincides with the measured one in Fig. 1(a). We can then calculate the dielectric properties from Gibbs free energy of BTS-*x* material system. The dielectric stiffness can be calculated from the second-order derivatives of the Gibbs free energy with respect to polarization,


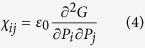


**Figure 5:**
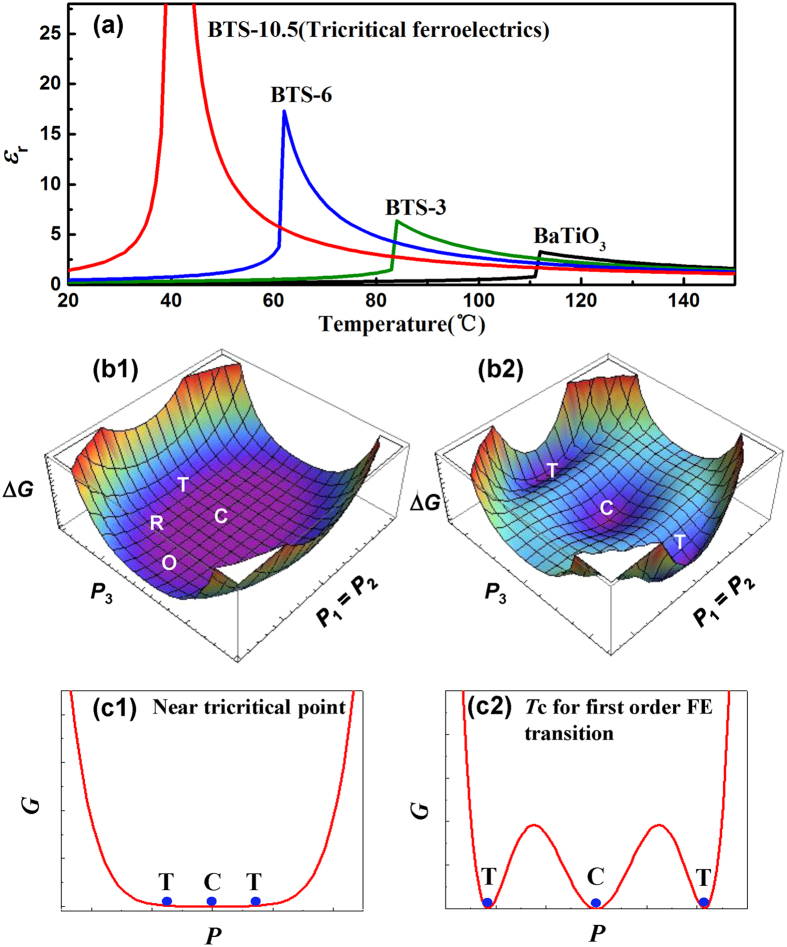
(**a**) The temperature-evolution of dielectric permittivity for different compositions calculated from the Landau model, and the tricritical phenomenon gives rise to the divergent permittivity in theory. (**b1**,**b2**) The comparison of Landau free energy profiles for near tricritical point and *T*c for first-order transition, and their 2D projections are shown in (**c1**,**c2**) respectively. The tricriticality causes the flat energy surface with vanishing barrier between different energy states and can thus facilitates large dielectric permittivity as well as energy density in low-field region.

The dielectric permittivity along the polar direction (*ε*_*r*_) can be calculated by the following equation^48^:


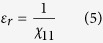


and the temperature-dependence for the reduced dielectric permittivity of each composition can be calculated by equation (4), (5), and is shown in Fig. 5(a). It can be seen that *ε*_*r*_ value has been greatly enhanced when it approaches the tricritical point, and the tricriticality will lead to divergent dielectric permittivity in the ideal case. However, this is seldom encountered in the experiments because of defects in sample. Instead, tricritical point composition exhibits a dielectric permittivity peak value maxima compared with other compositions. This gives the reason why large dielectric response has been achieved from the tricritical phenonmenon. We then compare the Gibbs free energy profiles for near tricritical point and conventional first order transition. Here we make *P*_*1*_ = *P*_*2*_ to show the (110) projection of the free energy profile. For the first order transition, the energy profile shows equal free energy for cubic (C) phase and tetragonal (T) phase (Fig. 5(b2)), but there exists energy barrier between the two phases (as shown in 2D profile in Fig. 5(c2)). However, in the vicinity of tricritical point, the energy profile shows flat free energy landscape (Fig. 5(b1)) and the energy barrier between C and T, O, R phases vanishes (as shown in 2D profile in Fig. 5(c1)). The vanishing energy barrier on the tricritical point can facilitate a large polarization change, and the material becomes easily polarized in the presence of the electric field, which can be ascribed as the reason for the large dielectric permittivity and thus high energy density at low electric field for ferroelectrics, which may find potential applications on low voltage energy storage devices.

It should be pointed out that there are two aspects that can be improved to further facilitate the future applications of tricritical ferroelectric ceramics. One is to enhance the breakdown strength for higher electric field application. The possible solutions may lie in, but not limited to the following approaches: (1) Fabricating thin film tricritical materials in order to enhance the breakdown strength^49^,^50^,^51^. (2) Adding glass additives to enhance the electric field endurance limit^52^,^53^. (3) Fabricating the polymer/tricritical ceramic composite material which exhibits higher breakdown strength^54^. Another one is to enhance the temperature stability. Despite of poor temperature stability as shown in Fig. 1(a) that is far from the requirement of commercialized capacitors (e.g. Z5U, Y5V, X7R *et al*.), large permittivity of such a material system can still be considered as the basis for further modification on facilitating temperature reliability. The possible solutions may lie in, but not limited to the following aspects: (1) adding T_C_ depressors to lower the sharpness of dielectric permittivity peak^55^. (2) producing relaxor ferroelectrics to enhances temperature stability due to the gradual change of polar nano-regions. (3) fabricating fine-grain ceramic that has good temperature reliability^56^.

## Summary

In conclusion, large dielectric response with ε_r_ = 5.4 × 10^4^ has been found in Ba(Ti_1-x%_Sn_x%_)O_3_ ferroelectric material system at the Curie temperature for *x* = 10.5, and relatively high energy density can reach to 30 mJ/cm^2^ at the electric field of 10 kV/cm. The optimal properties are proved to be caused by intrinsic effect under external field, which is responsible for phase transition. Further thermal analysis suggests that tricritical phenomenon occurs on the Curie temperature of *x* = 10.5, and the microstructure manifests itself as numerous of nanodomains with polarization inhomogeneity. A sixth-order thermodynamic modeling based on our experimental results suggests the flattening of free energy profile on tricritical point, and the easy altering of polarization in the presence of external electric field leads to large dielectric response and the associated energy density. Our finding can be found the potential applications on the low voltage energy storage devices such as the power sources of portable and wearable electronics.

## Method

A series of Ba(Ti_1-x%_Sn_x%_)O_3_ ceramics were prepared by using conventional solid-solution method starting from BaCO_3_, SnO_2_, TiO_2_ chemicals, and the powders were mixed and then calcinated at 1350 °C for 3 h. After shaping, the final ceramic specimens are obtained by sintering at 1450 °C for 3 h. Dielectric permittivity during phase transitions were detected by a LCR impedance analyzer equipped with a temperature chamber. The polarization (*P*)-electric field(*E*) hysteresis loops were measured by using ferroelectric workstation (Radiant techonology) at unipolar mode as well as bipolar mode. In order to monitor the phase transition behavior, thermal properties such as latent heat and specific heat were measured by differential scanning calorimeter (DSC) and Physical Property Measurement System (PPMS) respectively. Moreover, the microstructure origin of the specimens have been characterized by means of transmission electron microscope (TEM JEM 2100 F, JOEL) equipped with a heating specimen holder. Before TEM observation, the ceramic samples were prepared following the conventional TEM sample preparation procedure, which consists of mechanically grinding, ultrasonic cutting, and dimpling. Further ion polishing was performed to achieve the electron-transparent sample.

## Additional Information

**How to cite this article**: Gao, J. *et al*. Enhancing dielectric permittivity for energy-storage devices through tricritical phenomenon. *Sci. Rep.* **7**, 40916; doi: 10.1038/srep40916 (2017).

**Publisher's note:** Springer Nature remains neutral with regard to jurisdictional claims in published maps and institutional affiliations.

## Figures and Tables

**Table 1:** Dielectric coefficients for Ba(Ti_1-x%_Sn_x%_)O_3_ (BTS-*x*) ceramic system.

	BaTiO_3_	BTS-6	BTS-10.5	BTS-15	BTS-20
*Tc* (°C)	121.33	73.55	36.77	−3.80	−46.28
Dielectric permittivity at 0.1 kHz	1.066 × 10^4^	2.524 × 10^4^	5.364 × 10^4^	2.962 × 10^4^	2.148 × 10^4^
tan δ (%)	2.775	4.454	3.934	3.931	7.654
